# Filociclovir Is an Active Antiviral Agent against Ocular Adenovirus Isolates In Vitro and in the Ad5/NZW Rabbit Ocular Model

**DOI:** 10.3390/ph14040294

**Published:** 2021-03-26

**Authors:** Eric G. Romanowski, Islam T. M. Hussein, Steven C. Cardinale, Michelle M. Butler, Lucas R. Morin, Terry L. Bowlin, Kathleen A. Yates, Robert M. Q. Shanks, Regis P. Kowalski

**Affiliations:** 1 The Charles T. Campbell Ophthalmic Microbiology Laboratory, UPMC Eye Center, Department of Ophthalmology, University of Pittsburgh School of Medicine, Pittsburgh, PA 15213, USA; yateska@upmc.edu (K.A.Y.); shanksrm@upmc.edu (R.M.Q.S.); kowalskirp@upmc.edu (R.P.K.); 2 Microbiotix, Inc., Worcester, MA 01605, USA; ihussein@microbiotix.com (I.T.M.H.); scardinale@microbiotix.com (S.C.C.); mbutler@microbiotix.com (M.M.B.); lmorin@microbiotix.com (L.R.M.); tbowlin@microbiotix.com (T.L.B.)

**Keywords:** filociclovir, antiviral, eye, adenovirus, EKC, in vitro, animal model

## Abstract

Presently, there is no FDA- or EMA-approved antiviral for the treatment of human adenovirus (HAdV) ocular infections. This study determined the antiviral activity of filociclovir (FCV) against ocular HAdV isolates in vitro and in the Ad5/NZW rabbit ocular model. The 50% effective concentrations (EC_50_) of FCV and cidofovir (CDV) were determined for several ocular HAdV types using standard plaque reduction assays. Rabbits were topically inoculated in both eyes with HAdV5. On day 1, the rabbits were divided into four topical treatment groups: (1) 0.5% FCV 4x/day × 10 d; (2) 0.1% FCV 4x/day × 10 d; (3) 0.5% CDV 2x/day × 7 d; (4) vehicle 4x/day × 10 d. Eyes were cultured for virus on days 0, 1, 3, 4, 5, 7, 9, 11, and 14. The resulting viral eye titers were determined using standard plaque assays. The mean in vitro EC_50_ for FCV against tested HAdV types ranged from 0.50 to 4.68 µM, whereas those treated with CDV ranged from 0.49 to 30.3 µM. In vivo, compared to vehicle, 0.5% FCV, 0.1% FCV, and 0.5% CDV produced lower eye titers, fewer numbers of positive eye cultures, and shorter durations of eye infection. FCV demonstrated anti-adenovirus activity in vitro and in vivo.

## 1. Introduction

Human adenoviruses (HAdV) cause a variety of respiratory infections, conjunctivitis, gastroenteritis, and hemorrhagic cystitis [1]. A number of antiviral agents have demonstrated antiviral activity in vitro and antiviral efficacy in small human clinical trials [1,2]. Among these infections, HAdV ocular infections (epidemic keratoconjunctivitis (EKC), follicular conjunctivitis, and pharyngeal conjunctival fever) are the most common ocular viral infections worldwide [3,4]. At present, there is no FDA- or EMA-approved antiviral for the topical treatment of these ocular infections [5].

**Figure 1:**
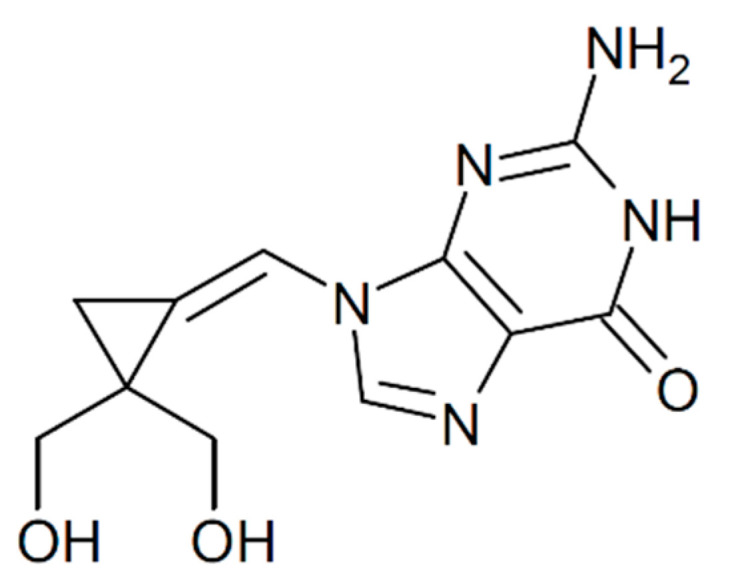
The chemical structure of filociclovir (FCV).

Filociclovir (FCV; cyclopropavir or MBX-400) is a novel methylene-cyclopropane nucleoside analog (Figure 1) that has demonstrated broad-spectrum antiviral activity against a number of herpesviruses including several that cause ocular disease, human cytomegalovirus (HCMV), varicella zoster virus (VZV), and Epstein–Barr virus (EBV) [6,7,8]. FCV has successfully completed systemic Phase I human safety studies [6,9] and is now entering Phase II clinical efficacy studies for the systemic treatment of HCMV viremia in transplant recipients [6]. FCV appears to inhibit HCMV replication using a complex mechanism of action that involves both the inhibition of the viral UL54 DNA polymerase and the UL97 kinase [7,10]. 

FCV was first shown to have antiviral activity against an American Type Culture Collection (ATCC) isolate of human adenovirus type 5 (HAdV5) [11]. A subsequent study demonstrated that FCV produced antiviral inhibitory activity against several HAdV isolates in vitro and in vivo in the treatment of an HAdV6 infection in the immunosuppressed Syrian hamster model [12]. That study reported that FCV was a potent and selective inhibitor of HAdV types 4–8 in vitro with 50% effective concentrations (EC_50_) in the range of 1.24–3.60 µM [12]. In the Syrian hamster model, a 10 mg/kg daily dose of FCV completely prevented mortality after an intranasal challenge of HAdV6 [12]. From other experiments presented in the article, the authors speculated that FCV inhibits HAdV replication through the inhibition of the adenovirus-encoded DNA polymerase by the triphosphate form of FCV [12]. 

While some adenovirus antiviral studies have been completed, currently, there have been no in vitro studies evaluating FCV against ocular isolates of HAdV or any in vivo antiviral studies in an HAdV ocular model. Furthermore, there have been no ocular toxicity or tolerability studies of topically instilled FCV to determine whether FCV is a viable candidate to be used in the eye. This led us to the current study for which the goals were to: (1) determine the in vitro antiviral activity of FCV against a panel of HAdV types and species that commonly infect the eye; (2) to determine the ocular toxicity and tolerability of topical 0.5% solution of FCV in normal rabbit eyes; and (3) to determine the antiviral efficacy of topical FCV in an ocular HAdV infection in the Ad5/NZW rabbit ocular model. The results of the study demonstrated that FCV produced in vitro antiviral activity against a panel of ocular HAdV types, was non-toxic to rabbit eyes, and demonstrated antiviral activity in the Ad5/NZW rabbit ocular model.

## 2. Results

### 2.1. In Vitro Antiviral Activity Assay (Plaque Reduction Assays)

The results from the in vitro antiviral plaque reduction assays (PRAs) are presented in Table 1 as the mean and standard deviations of the 50% effective concentration (EC_50_; concentration that inhibits plaque formation by 50%) (µM) from triplicate assays. The mean in vitro EC_50_ for FCV ranged from 0.50 to 4.68 µM, whereas those for CDV ranged from 0.49 to 30.3 µM across the panel of seven HAdV types tested. FCV produced lower EC_50_ for five of the seven HAdV types compared to CDV, which was used as an experimental positive control for antiviral activity [12,13].

### 2.2. Ocular Toxicity and Tolerability Study 

**Figure 2:**
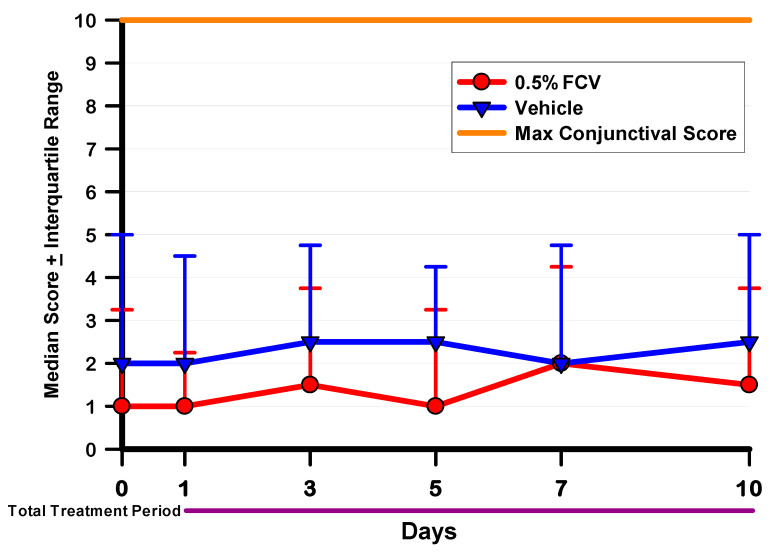
Median and interquartile ranges of the total conjunctival scores for 0.5% FCV and vehicle for each observation day in the ocular toxicity study. There were no significant differences between 0.5% FCV and the vehicle for any day (*p* > 0.05, K–W).

**Figure 3:**
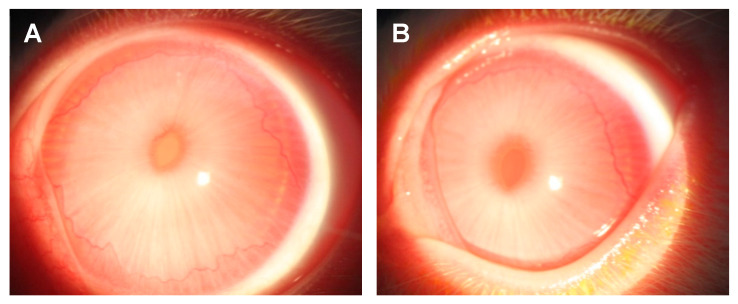
Representative photographs of eyes treated with (**A**) 0.5% FCV and (**B**) vehicle after 4 times daily dosing for 10 consecutive days in the ocular toxicity study.

The ocular toxicity and tolerability study evaluated 0.5% FCV compared to its vehicle using the modified MacDonald–Shadduck ocular scoring system during slit-lamp examination [14]. The naïve rabbits were topically treated in both eyes four times daily for 10 consecutive days. The topical 0.5% FCV and vehicle were well tolerated. There were no adverse behaviors demonstrated after instillation of either the 0.5% FCV or its vehicle at any time throughout the course of the study, indicating that the formulations were comfortable to the eyes. Regarding toxicity, neither treatment produced any signs of corneal toxicity (opacity, edema, corneal neovascularization, or corneal staining after fluorescein instillation). Minimal conjunctival signs (congestion (redness), chemosis (swelling), and discharge (tearing)) were noted before and during treatment in both groups. The scores from these three measures were added together to produce a total conjunctival score for each day and each eye. These data were analyzed using the Kruskal–Wallis (K–W) ANOVA with Dunn’s multiple comparisons test. The median ± interquartile ranges of the total conjunctival scores for each examination day are presented in Figure 2. The maximum total conjunctival score is 10. There were no significant differences in scores demonstrated between the two treatments for any examination day, nor were there any significant differences in scores over time (*p* > 0.05 K–W). Representative photographs of an eye from each treatment group following 10 days of treatment are presented in Figure 3. 

### 2.3. In Vivo Antiviral Activity Study 

**Figure 4:**
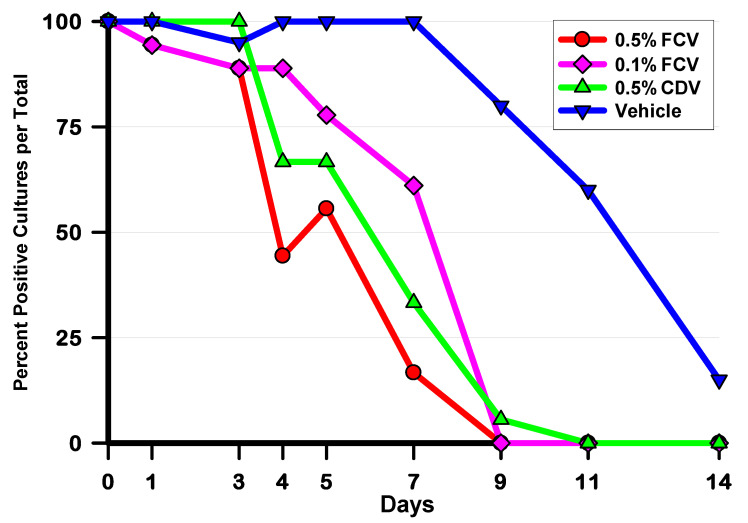
Percentages of HAdV5-positive cultures per total for each treatment group and culture day. Significant differences were demonstrated on the following days: **Day 4:** (0.5% FCV = 0.5% CDV < VEH; 0.5% FCV < 0.1% FCV); **Day 5:** (0.5% FCV = 0.5% CDV = 0.1% FCV < VEH); **Day 7:** (0.5% FCV, 0.5% CDV, 0.1% FCV < VEH; 0.5% FCV < 0.1% FCV); **Day 9:** (0.5% FCV = 0.1% FCV = 0.5% CDV < VEH); **Day 11:** (0.5% FCV = 0.1% FCV = 0.5% CDV < VEH). *p* ≤ 0.05, chi-Square or Fisher’s exact test (FET) (< is significantly fewer HAdV5-positive eyes per total).

The percentages of HAdV5-positive cultures per total for each culture day are presented in Figure 4. This is the most stringent of the viral outcome measures, as one HAdV5 plaque is considered a positive culture. Compared to the vehicle control, both 0.5% FCV and CDV significantly reduced the percentage of HAdV5-positive cultures per total on Days 4, 5, 7, 9 and 11, while 0.1% FCV demonstrated fewer HAdV5-positive cultures per total on Days 5, 7, 9, and 11 (*p* ≤ 0.026, chi-square or Fisher’s exact test (FET)). There were no differences between 0.5% FCV and CDV and 0.1% FCV and CDV on any day. However, 0.5% FCV significantly reduced the percentage of positive cultures per total compared to 0.1% FCV on Days 4 and 7 (*p* ≤ 0.006, chi-square).

**Figure 5:**
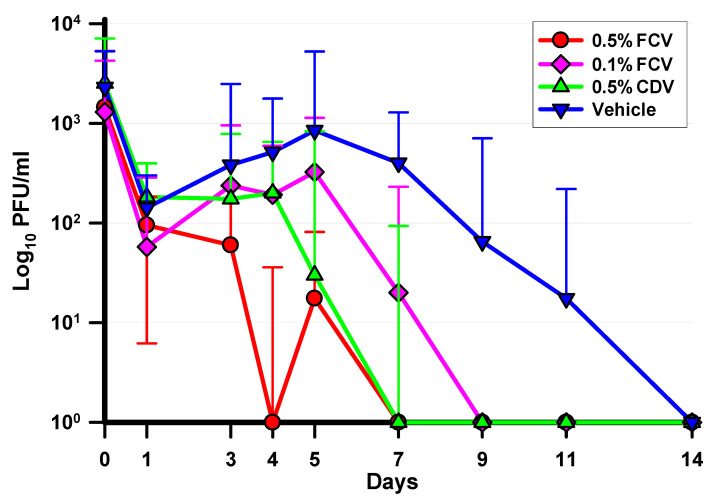
Log_10_ median ± interquartile ranges of HAdV5 titers for each treatment group and culture day. Significant differences were shown on the following days: **Day 1:** (0.5% FCV < 0.5% CDV); **Day 3:** (0.5% FCV < VEH); **Day 4:** (0.5% FCV < 0.1% FCV = VEH); **Day 5:** (0.5% FCV = 0.5% CDV < VEH); **Day 7:** (0.5% FCV = 0.5% CDV = 0.1% FCV < VEH); **Day 9:** (0.5% FCV = 0.1% FCV = 0.5% CDV < VEH); **Day 11:** (0.5% FCV = 0.1% FCV = 0.5% CDV < VEH). *p* ≤ 0.05, K–W. (< is significantly lower titers).

The Log_10_ median ± interquartile ranges of the daily ocular HAdV5 titers are presented in Figure 5. These data were analyzed using the Kruskal–Wallis ANOVA with Dunn’s multiple comparisons test. All tested antiviral formulations significantly decreased HAdV5 titers compared to the vehicle control, but to different degrees. Treatment with 0.5% FCV was the most active, significantly reducing HAdV5 titers on Days 3, 4, 5, 7, 9, and 11. Treatment with 0.1% FCV was less active, only reducing HAdV5 titers later in the infection on Days 7, 9, and 11. The activity of 0.5% CDV was in between the FCV groups, reducing titers on Days 5, 7, 9, and 11. On Day 4, 0.5% FCV had significantly lower titers than 0.1% FCV and a lower titer than 0.5% CDV on Day 1. There were no significant differences among the groups on any other days.

The duration of HAdV5 shedding for each eye was determined by the final day on which the eye had a positive HAdV5 culture. This outcome measure is presented as the median (± interquartile ranges) length of the infection in the eyes. Treatment with 5% FCV (5.0 ± 2.00 days), 0.1% FCV (7.0 ± 2.25 days), and 0.5% CDV (5.0 ± 3.25 days) significantly decreased the length of infection compared to the vehicle control (11.0 ± 2.00 days) (*p* ≤ 0.0002, K–W). There were no significant differences among the treatment groups. 

## 3. Discussion

Nucleoside analog antiviral agents have a long history of use in ophthalmology. The first antiviral approved by the FDA for human use (1963) was a topical treatment for herpetic epithelial keratitis, idoxuridine (IDU) [15,16]. Over subsequent years, many other nucleoside analog antivirals have been used to treat viral eye infections. Trifluridine (TFT), acyclovir (ACV), vidarabine (ara-A), and ganciclovir (GCV) have been used in the USA or Europe to topically treat herpetic epithelial keratitis caused by herpes simplex virus type 1 (HSV-1) [15]. Valacyclovir (VACV), ACV, and famciclovir have been used systemically to treat herpetic epithelial keratitis and to reduce local ocular toxicity produced by some of the topical agents [15]. CDV, GCV, and valganciclovir (VGCV) are used for systemic and local treatment of HCMV retinitis [17]. These nucleoside analog antivirals have demonstrated clinical efficacy and safety for nearly 60 years.

While there are approved antivirals available to treat ocular infections caused by herpesviruses, there are no FDA- or EMA-approved antivirals available to treat eye infections caused by adenoviruses. The nucleoside analogs CDV [13,18,19,20,21], zalcitabine (ddC) [21], and GCV [22] have been evaluated against adenovirus in vitro and in animal models of adenoviral ocular infection. Although the results of those studies demonstrated that these nucleoside analogs produced antiviral activity in those models, none of them have been approved for use in patients. Therefore, the search for a safe and effective nucleoside analog antiviral for adenovirus eye infections continues.

This search has led us to a promising new nucleoside analog, filociclovir. FCV has already been shown to possess in vitro and in vivo antiviral activity against HAdVs [11,12]. However, its antiviral activity against ocular isolates of HAdV, its ocular tolerability, and antiviral efficacy in animal models had not yet been determined. In the current study, we sought to answer these questions.

This study demonstrates that FCV exhibits antiviral activity against a panel of ocular HAdV isolates in the range of 0.5–5.0 µM. This is in the same range as the comparator antiviral CDV, with FCV demonstrating lower EC_50_ than CDV for five of seven isolates. These EC_50_ are similar to those produced in a previous anti-adenoviral study (1.24–3.60 µM) [12]. The in vitro evaluation of FCV cytotoxicity has been completed previously [12]. In this study, the 50% cytotoxic concentration (CC_50_) was found to be >100 or >150 µM depending on the cell line used [12]. The authors of this previous study concluded that the selectivity index (ratio of cytotoxicity over antiviral activity) of FCV was high for those HAdV types tested [12]. Since our study demonstrates similar EC_50_ values, the selectivity index of these additional HAdV types should also be considered high.

This panel of isolates represents HAdV types that are commonly associated with eye infections. Adenovirus types HAdV3, HAdV4, and HAdV7a are associated with follicular conjunctivitis and pharyngeal conjunctival fever, while HAdV8, HAdV19/64, and HAdV37 are associated with EKC, and HAdV5 is used in the Ad5 NZW rabbit ocular model. It is important to demonstrate broad-spectrum antiviral activity against the range of HAdV types and species as it has been previously reported that antivirals have variable activity across types and species of HAdVs [23]. Furthermore, we wanted to determine whether the antiviral activity demonstrated against HAdV5 was similar to the other common ocular adenovirus types so we could use HAdV5 as a surrogate for the other adenovirus species and types in the NZW rabbit ocular model. The results of the current in vitro study indicate that this is the case for FCV. In fact, HAdV5 produced the highest EC_50_ of the isolates tested (Table 1). Therefore, we conclude that the antiviral efficacy demonstrated against HAdV5 in vivo would translate to the other HAdV types.

The second goal was to determine the ocular toxicity and tolerability of FCV in rabbit eyes. We chose to evaluate 0.5% FCV and its vehicle in naïve rabbit eyes. The FCV and vehicle drops were well tolerated. No adverse behavior from the rabbits was seen after instillation of the drops four times daily for 10 consecutive days. This indicates that the formulations are comfortable to the eyes. The formulations were also non-toxic to the eyes during the treatment period. There were no corneal signs of toxicity noted in any of the eyes and only minor conjunctival scores. There were no significant differences in total conjunctival scores between the 0.5% FCV-treated eyes and the vehicle-treated eyes. In fact, the median scores were slightly lower for the 0.5% FCV on several examination days. There was no cumulative toxic effect of FCV over time since there was no significant increase in total conjunctival scores from Day 0 through Day 10. Histological analysis of the eyes showed no differences or abnormalities (data not shown), confirming the clinical data. Long-term and delayed toxicity after cessation of treatment was not evaluated in this study. These evaluations will be conducted in future studies.

Finally, the antiviral activity of FCV was evaluated in an ocular model of adenovirus infection. Our group has used the Ad5/NZW rabbit ocular model for several decades to evaluate potential antiviral agents for adenoviral ocular infection. In the current study, we evaluated two concentrations of FCV, 0.5% and 0.1%, using a standard treatment regimen of four times daily. We treated for 10 days to maximize the antiviral effect. We compared the efficacy to 0.5% CDV, twice daily for 7 days. This is the standard CDV treatment regimen that we have used in several studies [20,21,24,25]. Both concentrations of FCV and CDV demonstrated statistically significant reductions of ocular HAdV5 titers and shortened the length of the infection. Specifically, 0.5% FCV, 0.1% FCV, and 0.5% CDV significantly reduced the percentages of HAdV5-positive cultures per total, daily HAdV5 viral eye titers, and significantly shortened the duration of HAdV5 shedding (length of the infection). 

Treatment with 0.5% FCV produced antiviral activity early in the infection. HAdV5 eye titers were significantly reduced compared to the vehicle beginning on Day 3 and continued throughout the course of the study until being completely eliminated by Day 9, while 80% of the vehicle eyes were still shedding virus. By Days 4–5, around half of the eyes had the virus completely eliminated from the ocular surface. On Day 7, 83% of the eyes were completely cleared from virus compared with 0% cleared in the vehicle-treated eyes. This translated to a shortened median length of viral shedding by 55% (11 days for vehicle vs. 5 days for FCV). This is an important outcome. Shortening the viral shedding by more than half in conjunction with reducing the viral load in the eyes early in the infection could alleviate some patient suffering while potentially reducing or eliminating the formation of subepithelial corneal infiltrates that are associated with severe forms of adenovirus ocular infections, including EKC. 

Within the FCV groups, treatment with 0.1% FCV was not as effective as 0.5% FCV. Compared to the vehicle control, 0.1% FCV significantly reduced HAdV5 titers starting on Day 7 compared with Day 3 for 0.5% FCV. The application of 0.5% FCV significantly decreased HAdV5 eye titers on Day 4 compared with 0.1% FCV and also reduced the percentage of HAdV5-positive cultures per total on Days 4 and 7. Treatment with 0.5% FCV also shortened the median duration of shedding by two days compared to 0.1% FCV, although this difference was not significant. These results suggest that 0.5% FCV is more active than 0.1% FCV, making the antiviral effect of FCV concentration dependent.

In conclusion, FCV possesses antiviral in vitro activity against a panel of ocular HAdV types and species. It is tolerable and non-toxic when instilled into rabbit eyes and has antiviral activity against HAdV5 in the Ad5/NZW rabbit ocular model. This experiment is the first to demonstrate the in vivo antiviral activity of FCV against an experimental adenovirus ocular infection. Further research must be done to optimize the topical ocular FCV formulation, concentration, and treatment regimen and duration before human trials for adenoviral conjunctivitis are initiated. 

## 4. Materials and Methods

### 4.1. Viruses and Cells 

De-identified isolates from a clinical validation bank of human adenovirus types HAdV3, HAdV4, HAdV5, HAdV7a, HAdV8, and HAdV19/64 were recovered at the Charles T. Campbell Ophthalmic Microbiology Laboratory from patients presenting with typical adenoviral ocular disease. The use of these isolates in this study did not require Institution Review Board (IRB)/Ethics Committee approval because neither direct patient contact nor personal information were involved. The types of the isolates were determined using serum neutralization. HAdV19/64 was originally characterized as HAdV19, but subsequent studies have determined that HAdV19 is actually HAdV64 [26]. Therefore, for the purposes of this study, the isolate is designated HAdV19/64. No clinical isolates of HAdv37 were recovered, so the American Type Culture Collection (ATCC, Manassas, VA, USA) reference strain of HAdv37 was used. The rationale for the HAdV isolates and types chosen was that HAdV3 (Species B), HAdV4 (Species E), and HAdV7a (Species B) are common causes of follicular conjunctivitis and pharyngeal conjunctival fever, HAdV8 (Species D), HAdV19/64 (Species D), and HAdV37 (Species D) are major causes of EKC, and HAdV5 (Species C) is used in the Ad5/NZW rabbit ocular model. A549 human lung carcinoma cells were used to prepare the virus stocks, for the in vitro studies, and for the determination of ocular viral titers in the in vivo study.

### 4.2. Experimental Drugs 

FCV, dissolved in DMSO to 20 mM, was used for the in vitro antiviral assays. For the in vivo studies, 0.5% and 0.1% FCV along with their vehicle (10% [2-Hydroxpropyl]-β-cyclodextrin (Millipore Sigma, St. Louis, MO, USA), 0.2% cremophore (Millipore Sigma, St. Louis, MO, USA)) (VEH) were provided by Microbiotix, Inc. (Worcester, MA, USA) and were used for the in vivo studies. Cidofovir (CDV) for the in vitro and in vivo studies was prepared from the 7.5% injectable form of cidofovir (Cidofovir Injection, Heritage Pharmaceuticals Inc., East Brunswick, NJ, USA). CDV was prepared in the same vehicle and used as a comparator antiviral as it has demonstrated antiviral activity against adenoviruses in vitro [12,13] and in the Ad5/NZW rabbit ocular model [18,19,20,21,24,25].

### 4.3. Animals 

Female New Zealand White rabbits weighing 1.1–1.4 kg were obtained from Charles River Laboratories’ Oakwood Rabbitry. All animal studies conformed to the ARVO Statement on the Use of Animals in Ophthalmic and Vision Research. University of Pittsburgh IACUC approval (19106241) was obtained and all federal guidelines regarding animal experimentation were followed.

### 4.4. In Vitro Antiviral Activity Assay (Plaque Reduction Assay (PRA)) 

These studies were performed using 24-well multiplates (Costar 3526, Corning Inc., Kennebunk, ME, USA) containing A549 cell monolayers. One plate per virus strain per drug was used. The 24-well multiplates were inoculated with approximately 100 PFU/well of virus. After 3 h of adsorption, the inocula were removed from all wells. One milliliter of overlay media containing 0.001 µM, 0.01 µM, 0.1 µM, 1.0 µM, 10 µM, or 100 µM of FCV or CDV was added to 3 wells each. To the remaining 6 wells, 1 mL of overlay media without test drug was added. The plates were incubated at 37 °C in 5% CO_2_ until plaque formation was visible in the control wells. At that time, the media were removed, and the cells were stained and fixed with 0.5% gentian violet in formalin, and the number of plaques per well counted. Triplicate experiments were performed. The EC_50_ for each virus isolate, test drug, and trial were determined using fitted line plot regression analysis (Minitab, State College, PA, USA). The mean and standard deviations of the EC_50_ for FCV and CDV were determined for the three experiments.

### 4.5. Ocular Toxicity and Tolerability Study 

Six naïve NZW rabbits were divided into 2 topical treatment groups of 3 rabbits each. The first group received 0.5% FCV while the second group received the vehicle. Both groups were treated in both eyes four times daily for 10 days. The eyes were examined using a slit-lamp (Topcon, Tokyo, Japan) and graded using the modified MacDonald–Shadduck ocular scoring system [10] before treatment and on Days 1, 3, 5, 7, and 10 at least 1 h after the final dose. Total conjunctival scores were determined for each day and group and analyzed using the Kruskal–Wallis test (K–W) (Minitab, State College, PA, USA). Notations of adverse behavior of the rabbits after instillation were made. Rabbits were assessed for vocalization, immediate or delayed eye wiping, and/or hiding after instillation of drops. These behaviors can be indicators that the formulations may be irritating or that they sting.

### 4.6. In Vivo Antiviral Activity Study (Ad5 NZW Rabbit Ocular Model) 

This study was performed using a total of 37 rabbits. Following systemic anesthesia with 40 mg/kg ketamine and 4 mg/kg xylazine administered intramuscularly and topical anesthesia from 2 drops of 0.5% proparacaine, the rabbits were inoculated in both eyes with 50 µL (1.5 × 10^6^ PFU/eye) of HAdV5 following 12 cross-hatched strokes of a #25 sterile needle on each cornea. Twenty-four hours later, rabbits were randomly assigned to one of four topical treatment groups: (1) 0.5% FCV: 4x per day for 10 days (*n* = 9); (2) 0.1% FCV: 4x per day for 10 days (*n* = 9); (3) 0.5% CDV: 2x per day for 7 days (*n* = 10); and (4) vehicle: 4x per day for 10 days (*n* = 9). The rabbits were treated topically in both eyes according to the above treatment regimens. Ocular cultures were performed on days 0, 1, 3, 4, 5, 7, 9, 11, and 14 after inoculation and at least 1 h after the final dose of antiviral. The conjunctival and corneal surfaces were cultured after topical anesthesia with 0.5% proparacaine using dacron-tipped applicators. The swabs were placed into tubes containing 1 mL of tissue culture media and were frozen at −75 °C pending the determination of HAdV5 titers.

### 4.7. Determination of Ocular Viral Titers (Plaque Assay) 

The ocular culture samples to be titered were thawed, diluted, and inoculated onto A549 cell monolayers in 24-well multiplates. Following adsorption for 3 h, 1 mL of overlay media was added to the wells. After 7 days of incubation at 37 °C in 5% CO_2_, the media were removed and cells were stained and fixed with 0.5% gentian violet in formalin, and the number of plaques per well counted. The viral titers were then calculated and expressed as plaque-forming units per milliliter (PFU/mL). 

### 4.8. Statistical Analyses 

Ocular titer and toxicity data were analyzed using Kruskal–Wallis ANOVA with Dunn’s multiple comparisons (GraphPad Prism, San Diego, CA, USA), and chi-square (Minitab, State College, PA, USA), or Fisher’s exact test (FET) (https://www.graphpad.com/quickcalcs/contingency1/, accessed on 1 April 2020). Significance was established at the *p* ≤ 0.05 confidence level.

## Figures and Tables

**Table 1:** Mean and standard deviations of EC_50_ from 3 plaque reduction assay (PRA) trials.

Virus	Filociclovir	Cidofovir
AdV3	0.78 ± 0.21 µM	5.78 ± 1.70 µM
HAdV4	4.31 ± 0.28 µM	8.71 ± 1.40 µM
HAdV5	4.68 ± 0.29 µM	30.3 ± 22.0 µM
HAdV7a	2.12 ± 2.59 µM	1.81 ± 2.33 µM
HAdV8	0.50 ± 0.08 µM	0.49 ± 0.03 µM
HAdV19/64	1.86 ± 2.58 µM	4.09 ± 3.71 µM
HAdV37	3.53 ± 2.67 µM	3.96 ± 6.22 µM

## Data Availability

The data reported in this study are available in this manuscript.
